# The characterization, renoprotection and antioxidation of enzymatic and acidic exopolysaccharides from *Hypsizigus marmoreus*

**DOI:** 10.1038/s41598-018-20440-y

**Published:** 2018-02-01

**Authors:** Min Liu, Yufei Lan, Chengye Tian, Yongfa Zhu, Hui Liu, Wenshuai Wang, Weiru Liu, Jianjun Zhang, Le Jia

**Affiliations:** 1 College of Life Science, Shandong Agricultural University, Taian, 271018 P.R. China; 2 Taian Academy of Agricultural Sciences, Taian, 271000 P.R. China; 3 The Comprehensive Supervision and Enforcement Bureau of Sanitation and Family-planning of Taian, Taian, 271000 P.R. China; 4 The Second High School of Taian, Taian, 271018 P.R. China

## Abstract

The present work was designed to investigate the characterization, as well as the antioxidation and renoprotection in streptozocin (STZ)-induced diabetic mice, of exopolysaccharides (EPS) and the enzymatic-EPS (EEPS) and acidic-EPS (AEPS) hydrolysates, which were separated from the fermentation liquor of *Hypsizigus marmoreus*. Animal results demonstrated that EPS, EEPS and AEPS had potential antioxidant and renoprotective effects, especially EEPS. Additionally, they were the most effective, reflecting increases in superoxide dismutase (SOD), glutathione peroxidase (GSH-Px), catalase (CAT), total antioxidant capacity (T-AOC), and albumin (ALB) of 168.33%, 124.8%, 268.17% 179.49%, and 68.71%, respectively, and decreases in the contents of malondialdehyde (MDA), lipid peroxide (LPO) and levels of serum urea nitrogen (BUN) and creatinine (CRE) by 70.58%, 58.43%, 23.97% and 29.60%, respectively, at a dose of 800 mg/kg compared to those of model mice. Three polysaccharides ameliorated the histopathological alterations which were observed in the kidney of diabetic mice. Furthermore, the characterization of polysaccharides had been expressed. These findings indicated that the EEPS from *H*. *marmoreus* possesses more effective renoprotection and antioxidation effects and provided insight into its potential clinical values on preventing diabetes.

## Introduction

Currently, chemical-synthetic medicaments, which are widely used clinically in curing many diseases, can cause serious cellular damage in the intracorporeal organs, including the heart, liver, kidney, and pancreas, and previous literature has indicated that these chemicals can be metabolically activated into highly reactive free radical compounds, which can induce oxidative stress, resulting the aggravation of disease progress^1^. Internationally, to keep away from these negative effects, many researchers have committed themselves to exploring natural and non-toxic substrates as conventional and effective clinical medicine^2^. The macrofungi of mushrooms, the most popular natural food, owing to its special mouthfeel and abundant nutrition, have gained increasing academic attention. They were widely used in identifying innovative drugs due to their vast bioactive compounds, such as proteins, polysaccharides, helvolic acid, and p-terphenyls. Additionally, these mushrooms have potential effects as antibacterial, anti-tumor^3^, antioxidant compounds, in protection against DNA damage^4^, and as neuroprotective compounds^5^. Numerous literature has demonstrated that polysaccharides, the most varied nutrient dense and abundant substances, have potential biological properties, such as the antioxidants of *Tricholoma mongolicum Imai*^6^, the antitumor compounds of *Lentinus edodes*^7^, and the anti-hyperlipidemic compounds of *Termitomyces albuminosus*^8^. In this regard, the polysaccharides from *H. marmoreus*, one of the most widely used traditional Chinese medicines and nutritional mushrooms, have attracted much attention because of its multiple pharmacological effects, including as antioxidant, anti-tumor, antiviral compounds and for their immunomodulating properties^9^. However, previous studies have focused their attentions on mycelia/intracellular and fruiting-body polysaccharides. However, there is scarce literature about exopolysaccharides/extracellular polysaccharides (EPS). Interestingly, Chen *et al*.^10^ indicated that EPS, a class of high-value biopolymers extracted from fermentation broth, have superior industrial application owing to their higher yields and related procedures that are less time-consuming and have less chance of contamination.

Diabetes mellitus (DM), the most common cause of death worldwide that is characterized by hyperglycemia, can lead to impaired metabolic functions of carbohydrates, lipids and proteins, inducing DM complications such as organ damage^11^. Streptozotocin (STZ), which is commonly employed in establishing diabetic animal models, can cause organ damage which is analogous to human symptoms^12^. Although the exact mechanism of STZ-induced toxicity is not well understood, several studies have shown the inevitable relationship between STZ-induced oxidative stress and kidney damage^13^. Experimentally, STZ can generate high amounts of reactive oxygen species (ROS), including superoxide anion, hydroxyl, alkoxyl and peroxyl radicals, and stimulate organic lipid peroxidation^14^, leading to serious oxidative stress. Notably, our previous studies have demonstrated organic dysfunction associated with STZ-induced toxicity that can be attenuated by the treatment with polysaccharides from mushrooms^11^.

The aim of the present work was designed to evaluate the renoprotective and antioxidant effects of EPS and its two hydrolysates, enzymatic-EPS (EEPS) and acidic-EPS (AEPS), from the fermentation broth of *H. marmoreus* in STZ-induced diabetic mice to better understand possible anti-diabetes mechanism and their health benefits in food and the pharmaceutical industry, indicating that the polysaccharides could be developed as valuable functional foods for clinical diabetes treatments. Furthermore, the exploration of fermentation liquor and the utilization of fermentation liquor-derived and value-added products, seem to be significant.

## Results

### Body weights and glucose (GLU) levels

As can be noted in Table 1, in the pretreatment condition, no significance was shown in both the body weights and GLU levels between the mice of the model control group (MC) groups and dosage groups (*P *> 0.05). Additionally, the significant increase in the GLU levels in MC mice compared with the normal control group (NC) mice indicated the successful hyperglycemia model. After two weeks, the body weight of the NC mice was significantly higher than those of the MC mice (diabetic mice treated with distilled water) (*P* < 0.05), and the GLU level of the NC mice was also lower than those of the MC mice (*P* < 0.05). Interestingly, for mice in the high-dose (800 mg/kg) EEPS group, the body weight was close to the normal group and increased by 41.80% compared with the MC mice. Meanwhile, the GLU level in the experiment group treated with EEPS at dose of 800 mg/kg decreased by 50.38% compared to those of the MC mice.

**Table 1:** Effects of EPS, EEPS, and AEPS on the body weights and GLU levels in the STZ-induced diabetic mice.

	Groups	Body weight (g)		GLU levels (mM)	
		Pre-treatment	Post-treatment	Pre-treatment	Post-treatment
	NC	20.06 ± 1.79^d^	35.93 ± 2.16^a^	4.38 ± 0.28^f^	4.32 ± 0.20^f^
	MC	19.92 ± 2.01^d^	23.61 ± 1.95^cd^	14.41 ± 0.83^a^	14.53 ± 0.69^a^
	PC	20.10 ± 1.82^d^	33.27 ± 2.04^a^	14.42 ± 0.79^a^	7.02 ± 0.41^e^
800 mg/kg	EPS	20.16 ± 2.15^d^	28.27 ± 2.07^ab^	14.40 ± 0.65^a^	7.83 ± 0.64^de^
	EEPS	19.93 ± 2.12^d^	33.48 ± 2.08^a^	14.46 ± 0.59^a^	7.21 ± 0.48^e^
	AEPS	19.93 ± 1.75^d^	30.06 ± 1.92^a^	14.44 ± 0.74^a^	7.44 ± 0.54^e^
400 mg/kg	EPS	20.11 ± 1.92^d^	27.06 ± 2.19^b^	14.42 ± 0.81^a^	9.05 ± 0.67^c^
	EEPS	19.82 ± 2.08^d^	30.63 ± 2.15^c^	14.46 ± 0.72^a^	8.16 ± 0.73^d^
	AEPS	20.01 ± 2.26^d^	28.42 ± 2.04^ab^	14.41 ± 0.53^a^	8.20 ± 0.57^d^
200 mg/kg	EPS	19.84 ± 1.84^d^	24.98 ± 1.94^c^	14.49 ± 0.72^a^	9.88 ± 0.85^b^
	EEPS	19.80 ± 1.97^d^	27.94 ± 2.07^b^	14.44 ± 0.82^a^	9.24 ± 0.69^b^
	AEPS	19.81 ± 2.16^d^	26.21 ± 2.14^b^	14.59 ± 0.69^a^	9.54 ± 0.68^b^

The values are reported as the means ± SD (n = 10). Bars with the same letter are not significantly different (*P* < 0.05).

### Histopathological analysis

**Figure 1:**
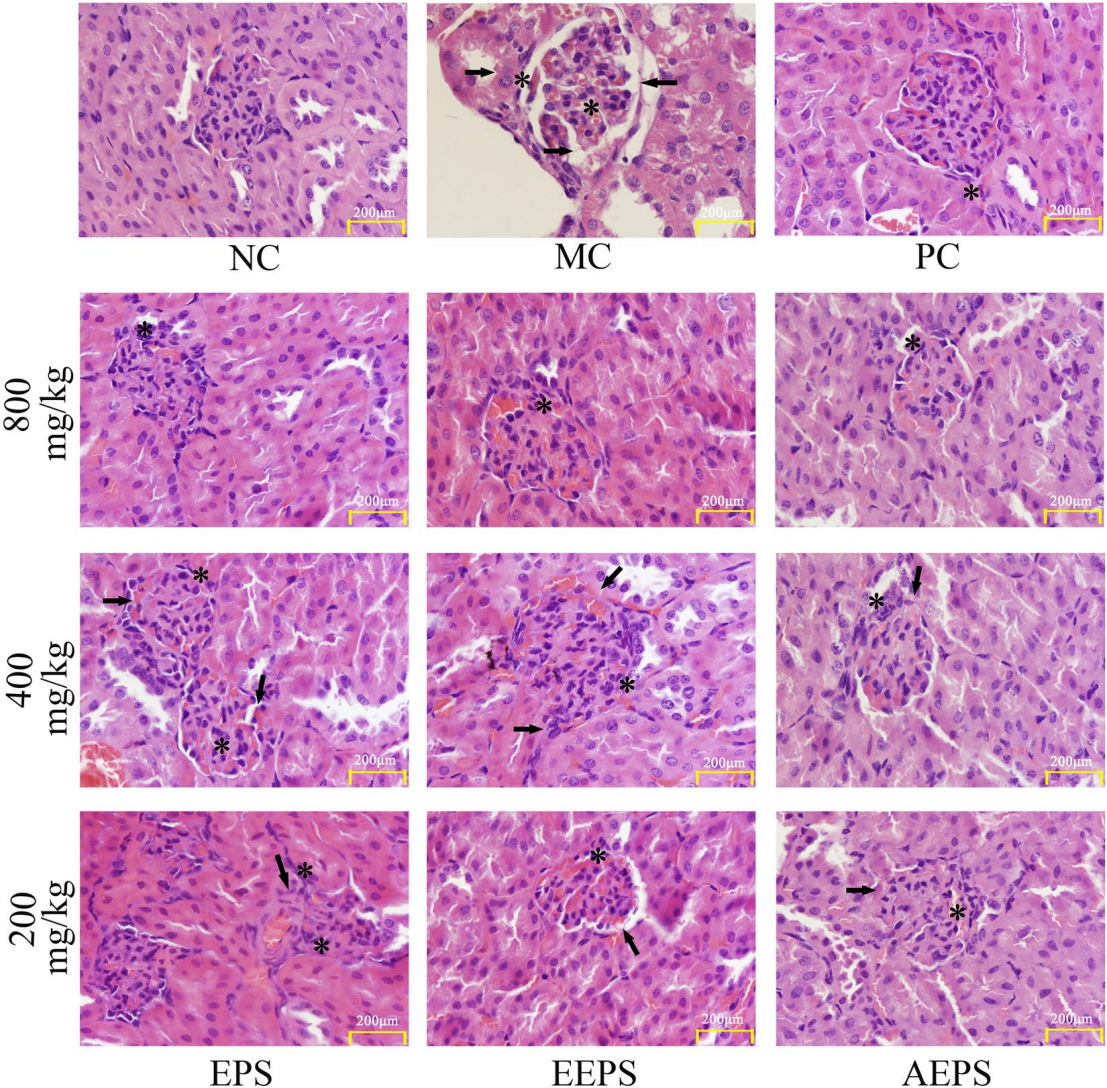
Effects of EPS, EEPS, and AEPS on kidney tissue damage in NC, MC, PC, and treated diabetic mice with EPS, EEPS, and AEPS at doses of 800 mg/kg, 400 mg/kg and 200 mg/kg (HE staining, magnification ×600, *cellular degeneration;  glomerulus destruction).

Obviously, the kidney damage in diabetic mice could also be confirmed by histopathological observations (Fig. 1), indicating that kidney damage was successfully established by STZ injection. In the MC group, diabetic mice exhibited serious glomerular degeneration and renal lesions reflected by inflammatory infiltration, extracellular matrix deposition, and glomerular basement membrane distortion, while the normal mice showed an integrated glomerulus and intact glomerular basement membranes. This was especially the case for the histopathological damage, which can be relieved in EEPS treatment mice at a dose of 800 mg/kg, and similar remission has been shown in the positive control (PC) group mice (Fig. 1).

### Effects of EPS, EEPS, and AEPS on kidney

**Figure 2:**
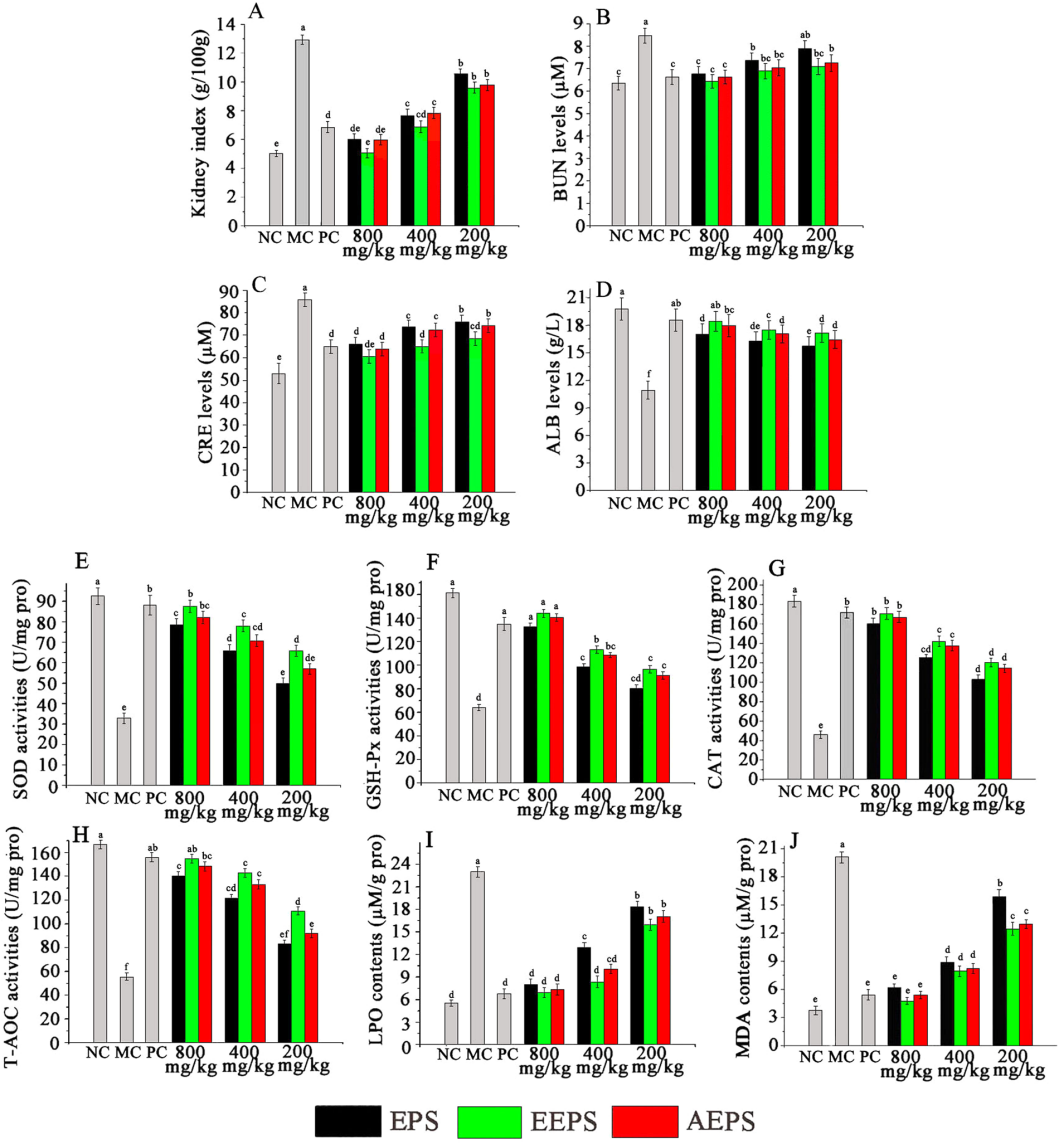
Effects of EPS, EEPS and AEPS on (**A**) kidney index, (**B**–**D**) serum analysis, (**E–H**) hepatic enzymatic analysis and (**I**,**J**) lipid peroxidation. The values are reported as means ± SD (n = 10). Bars with the same letter are not significantly different (*P* < 0.05).

As seen in Fig. 2A, significant increase in the kidney index observed in the MC group in comparison with the NC group. Presently, after the treatment with EEPS, AEPS and EPS, the kidney index increase observed in the MC group was alleviated (Fig. 2A), especially the treatment of EEPS at the dose of 800 mg/kg.

As displayed in Fig. 2B,C, the serum urea nitrogen (BUN) and creatinine (CRE) were 8.47 ± 0.33 mM and 85.84 ± 3.00 µM, respectively, in the diabetic mice (MC group), which were markedly increased in comparison with the NC mice (6.35 ± 0.30 mM and 52.95 ± 4.51 µM), while the albumin (ALB) declined in MC (10.93 ± 1.01 g/L) compared with NC mice (19.76 ± 1.20 g/L). Interestingly, three polysaccharides at different doses could ameliorate the biochemical enzyme activities of the STZ-induced mentioned above in comparison with the MC mice, especially the EEPS at a dose of 800 mg/kg, which decreased the BUN and CRE by 23.97% and 29.60%, respectively, compared to those of the MC group. Meanwhile, BUN and CRE decreased by 21.72% and 25.59%, respectively after treatment with AEPS, and BUN and CRE also decreased by 19.95% and 23.25%, respectively, by treatment with EPS at the same dose (800 mg/kg), while the ALB levels were increased by 68.71%, 64.23% and 55.72% in EEPS, AEPS and EPS treatment, respectively, in mice at a dose of 800 mg/kg. The enzyme activities contained superoxide dismutase (SOD), glutathione peroxidase (GSH-Px), catalase (CAT), total antioxidant capacity (T-AOC), as well as the contents of lipid peroxide (LPO) and malondialdehyde (MDA), which were observed in Fig. 2E–J. Compared with the mice in the NC group, the significant decrease in the SOD, GSH-Px, CAT and T-AOC activities and significant increase in the LPO and MDA contents in the MC group indicated that the kidney had been seriously damaged (*P* < 0.05). Obviously, the treatment of EEPS, AEPS and EPS expressed concentration-dependent patterns at the tested concentrations with increasing activities of SOD, GSH-Px, CAT and T-AOC, as well as decreasing contents of LPO and MDA (*P* < 0.05). Especially, the activities of SOD, GSH-Px, CAT and T-AOC were increased by 168.33%, 124.8%, 268.17% and 179.49%, respectively, and the contents of LPO and MDA were decreased by 70.58% and 58.43%, respectively, compared to those of the MC mice with EEPS at a dosage of 800 mg/kg. The PC group mice treated with glibenclamide also expressed remarkable increase in the GSH-Px, CAT, SOD and T-AOC activities and significant decline in the MDA and LPO contents compared with the MC mice.

### Effects of EPS, EEPS, and AEPS on serum lipid levels

**Figure 3:**
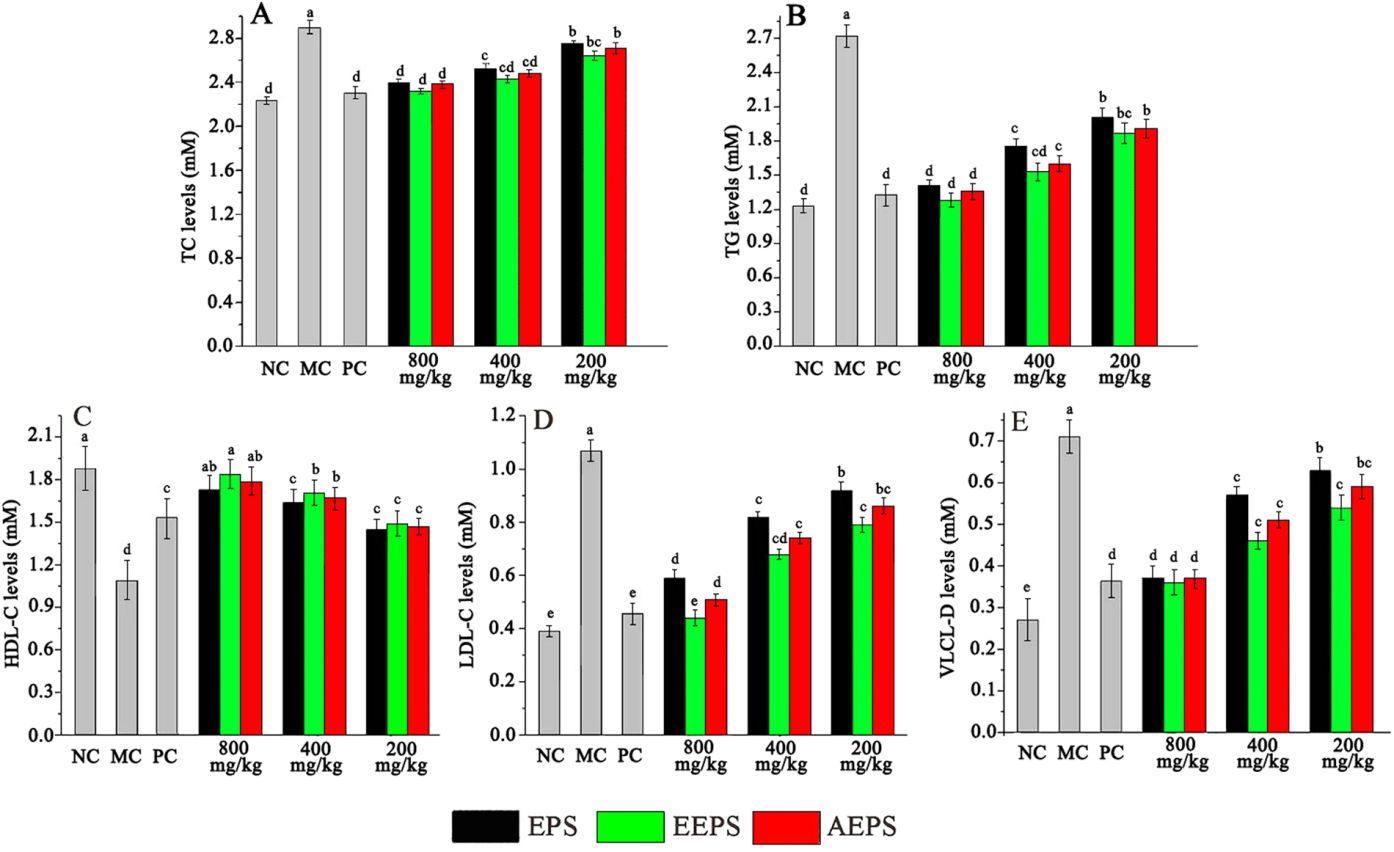
Effects of EPS, EEPS and AEPS on lipid metabolism. (**A**) TC levels, (**B**) TG levels, (**C**) HDL-C levels, (**D**) LDL-C levels, and (**E**) VLDL-C levels. The values are reported as means ± SD (n = 10). Bars with the same letter are not significantly different (*P* < 0.05).

Figure 3 shows that the serum total cholesterol (TC) levels, triglyceride (TG) levels, high density lipoprotein cholesterol (HDL-C) levels, low-density lipoprotein cholesterol (LDL-C) levels, and very low-density lipoprotein cholesterol (VLDL-C) levels in diabetic mice were significantly higher (*P* <  0.05), while the HDL-C level was significantly lower than those in the MC groups (*P* < 0.05), indicating the disordered circulatory lipoproteins. After two weeks of gavage administration, the TC, TG, LDL-C, and VLDL-C levels were decreased significantly (*P* < 0.05), and the HDL-C levels were increased significantly in the dosage groups compared to those in the MC groups (*P* < 0.05, Fig. 3), indicating that the three polysaccharides had potential protective effects. In EEPS treated mice at 800 mg/kg, the TC, TG, LDL-C, and VLDL-C levels were decreased by 20%, 52.94%, 58.88% and 49.29%, respectively, and the HDL-C level was increased by 68.81% compared to those in the MC group. Similar conclusions could be drawn from the positive control mice treated with glibenclamide, suggesting the protective effects of positive medicine.

### Acute toxicity study

During the whole period of treatment with EEPS, AEPS and EPS, even at a dose of 4000 mg/kg, all the mice showed normal activities and did not show any clinical signs of toxicity. Furthermore, no death was observed either originally or finally, indicating that these three polysaccharides were all practically non-toxic substances^15^.

### The primer analysis of characteristics

High-performance liquid chromatography (HPLC) chromatograms indicated that the Mw (weight-average molecular weight), Mn (number-average molecular weight) and Mz (z-average molecular weight) of EEPS were 2.14 × 10^3^, 1.12 × 10^3^ and 1.39 × 10^3^ Da, while the Mw, Mn and Mz of EPS were 1.07 × 10^3^, 1.15 × 10^3^ and 1.14 × 10^3^ Da, and the Mw, Mn and Mz of AEPS were 1.20 × 10^3^, 1.95 × 10^3^, 1.41 × 10^3^ Da, respectively.

**Figure 4:**
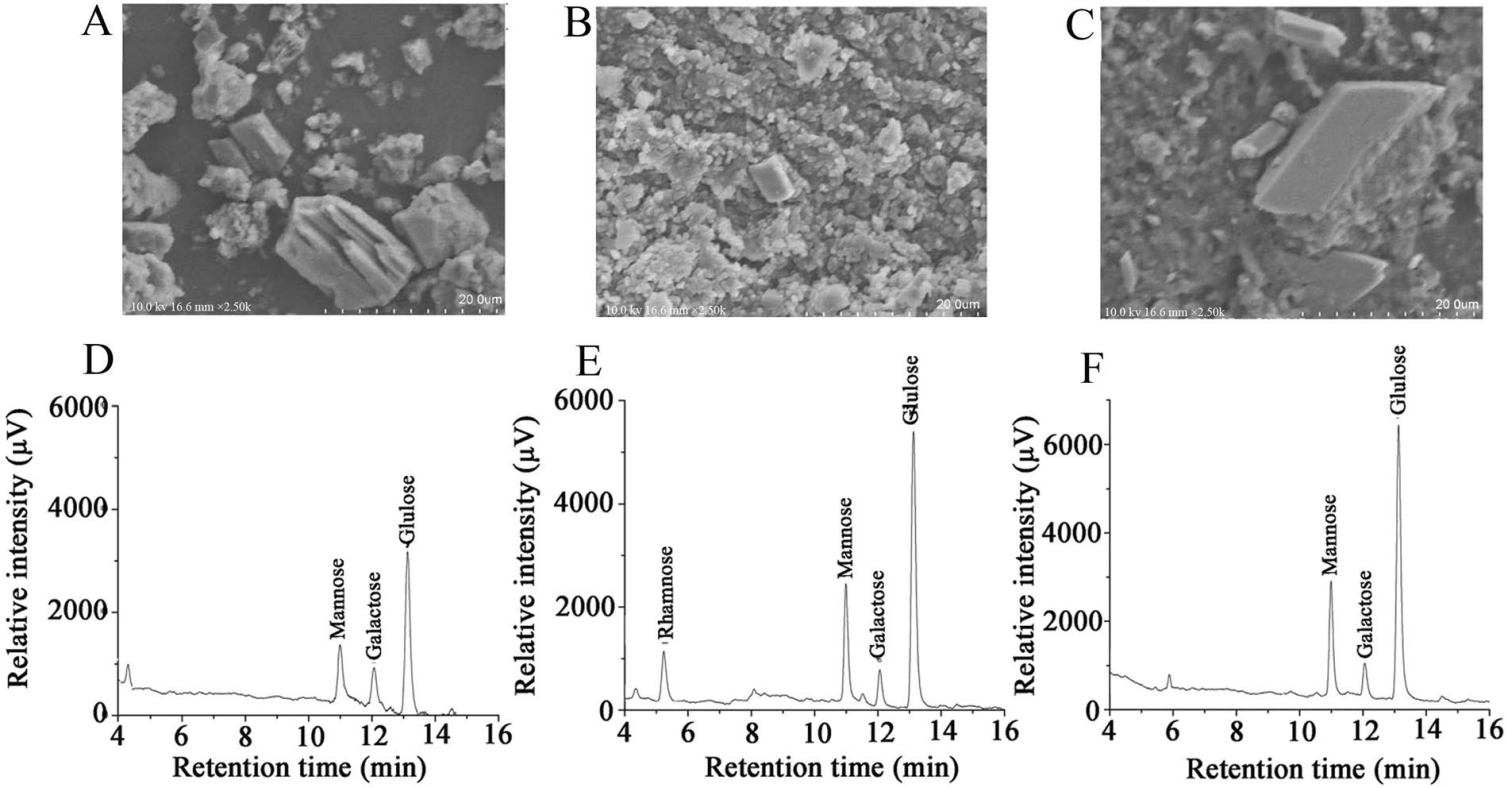
The SEM analysis of (**A**) EPS, (**B**) EEPS and (**C**) AEPS, and GC spectra of (**D**) EPS, (**E**) EEPS and (**F**) AEPS.

Scanning electron microscope (SEM) images of EPS, EEPS and AEPS are shown in Fig. 4A–C. Under 25000 × magnification, EPS showed an integrated surface with several regular shapes (Fig. 4A). Different from EPS, the microstructure of EEPS and AEPS presented an irregular stratified structure, and many holes with a non-uniform size were bestrewed on its surface (Fig. 4B,C).

The monosaccharide compositions of EPS, EEPS and AEPS were identified by comparing the retention time with standard monosaccharides (Fig. 4D–F) by gas chromatography (GC) analysis. Obviously, EEPS contained rhamnose, mannose, galactose and glucose with a molar ratio of 1.1:2.3:1.0:6.4 (Fig. 4E), while EPS and AEPS were both composed of mannose, galactose and glucose with different molar ratios of 1.1:1.0:2.3 (EPS, Fig. 4D) and 2.5:1.0:6.9 (AEPS, Fig. 4F), respectively.

**Figure 5:**
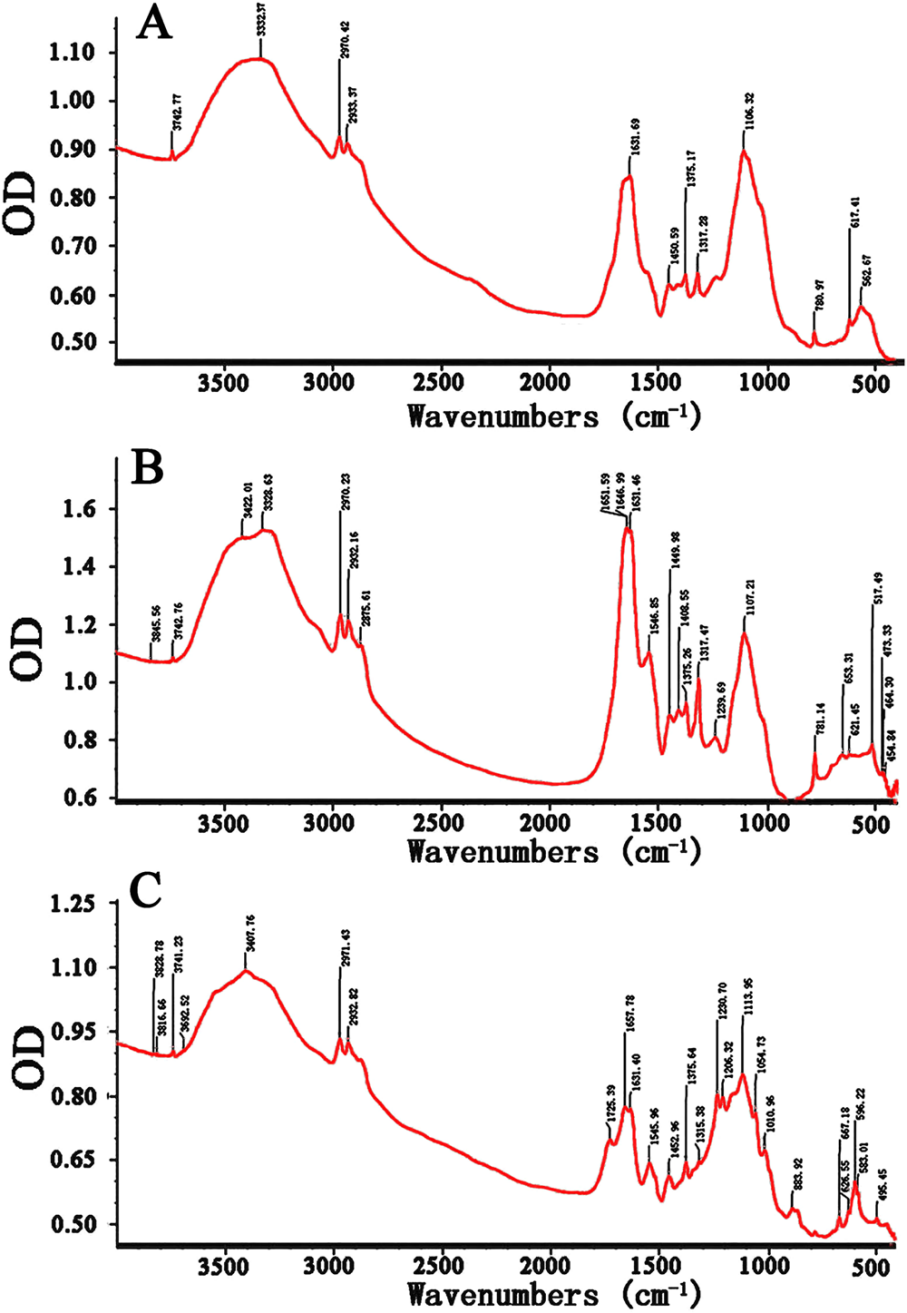
The FT-IR spectra of (**A**) EPS, (**B**) EEPS and (**C**) AEPS.

Fourier transform infrared (FT-IR) spectroscopy revealed a typical major broad peak of hydroxyl stretching vibration around at 3332.37 cm^−1^ (EPS), 3422.01 cm^−1^ (EEPS), and 3407.76 cm^−1^ (AEPS), respectively. The three polysaccharides showed bands around 2900–3000 cm^−1^ for C-H stretching vibration. The bands in the region of 1631.69 cm^−1^ (EPS), 1631.46 cm^−1^ (EEPS) and 1631.40 cm^−1^ (AEPS) indicated the presence of C=O groups of amide stretching vibration (-CO-NH_2_). This demonstrated that the small amounts of protein in the samples may be sugar-binding proteins. The bands at 1450.59, 1449.98 and 1452.96 cm^−1^ corresponded to the characteristic C-O (-COOH) stretching vibration. The bands at 1375.17, 1375.26 and 1375.64 cm^−1^ corresponded to the C-H variable angle vibration in EPS, EEPS and AEPS (Fig. 5A–C). The band at 1239.69 cm^−1^ indicated the O-H (-COOH) variable angle vibration in EEPS. Additionally, the fingerprint region between 650 and 1350 cm^−1^ was usually associated with the stretching vibrations of C-C and C-O, as well as the bending mode of the C-H band. It had previously been suggested as a promising area for the analysis of structural conversions, especially the region of 980–1170 cm^−1^, which was sensitive to OH groups and could significantly affect the main band positions. As shown in Fig. 5, the bands in the 1010.96 cm^−1^ (AEPS) region were due to C-O and O-H stretching vibrations, suggesting that both pyranose rings existed in AEPS^16–18^.

**Figure 6:**
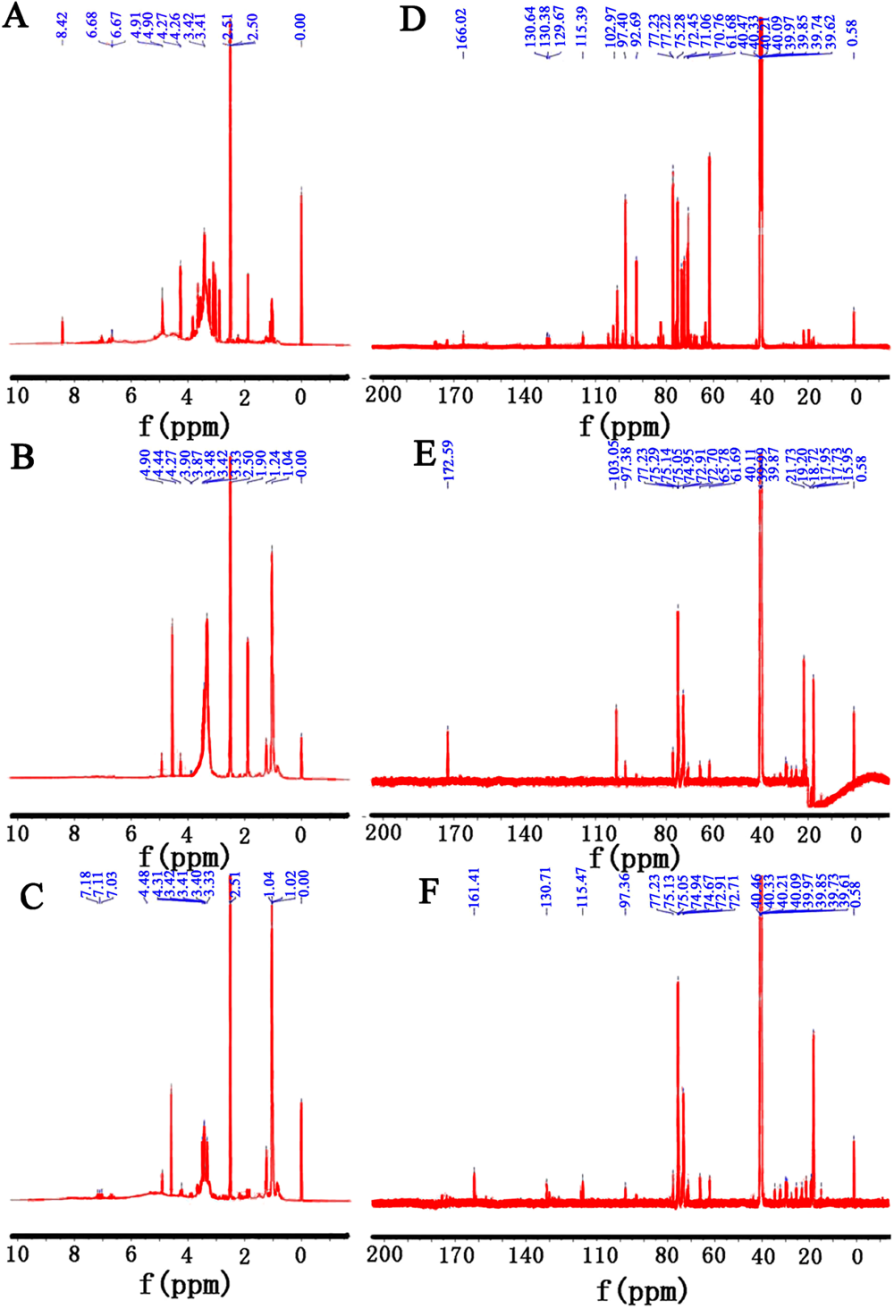
The ^1^H NMR spectra of (**A**) EPS, (**B**) EEPS and (**C**) AEPS, and ^13^C NMR spectra of (**D**) EPS, (**E**) EEPS and (**F**) AEPS.

The ^1^H nuclear magnetic resonance spectroscopy (NMR) spectrum of EPS showed four signals at 4.91, 4.90, 4.27 and 4.26 ppm corresponding to anomeric protons (Fig. 6A). In this spectrum, the 4.1 and 4.6 ppm signals corresponded to signals obtained for β glucan (Fig. 6A). Similarly, the ^13^C NMR spectrum contained four anomeric carbon signals from 92.69 to 104.61 ppm (Fig. 6D). The results suggested that EPS contained both α- and β-anomeric configurations. The ^1^H spectra of EEPS and AEPS showed a chemical shift in the anomeric region at 4–6 ppm (Fig. 6B,C). Similarly, the ^1^H spectrum of the polysaccharides exhibited a set of wide and intense signals (3.0–4.0 ppm) due to the CH_2_-O and CH-O groups of the sugars, while the chemical shifts from 3.2 to 4.1 ppm were assigned to the H-2 to H-6 protons. Furthermore, the regions between 1.4 and 2.5 were also observed to relate to the glucan-protein structure. Figure 6E,F showed the ^13^C NMR spectra of EEPS and AEPS from *H. marmoreus*. The presence of glucose could be observed by signals at 102.97 and 103.15 ppm^19,20^.

## Discussion

Pharmacologically, toxicity studies have always been considered as vital evidence during drug development. Different drugs have been synthesized for the treatment of DM; however, many synthetic drugs have a number of serious side effects^1^. With comparatively low side effects, polysaccharides from mushroom were used as potential medicines to open new avenues for the treatment of various diseases due to their biological activities^6–8^. Many investigators focused their academic attention on the study of antioxidation from either the fruiting body or mycelia of various medicinal fungi. However, there still been poor publication of reports focusing on the toxicity and biological activities of polysaccharides with the necessary modifications. It was necessary determine whether the biological activities will remain when EEPS and AEPS are orally administered. Hence, our present work was designed to investigate the renoprotective effects of EPS and their hydrolysates (EEPS and AEPS) from *H. marmoreus* in mice against oxidative stress induced by STZ injection.

In recent years, mushroom polysaccharides have been confirmed to possess higher antioxidant activities in protecting against lots of diseases induced by ROS^13^. DM, a group of metabolic diseases accompanied by organ damage, had been reported to be a complicated disease related to oxidative stress and have become currently the most common danger, greatly threatening human health^21^. Development of therapies to prevent the generation of free radicals may influence the progression of oxidative organ damage induced by STZ. Although the exact mechanisms of STZ-induced toxicity remain poorly understand, previous studies suggested that lipid peroxidation and free radical formation had devastating roles in the development and progress of diabetes^11^. The possible mechanism may be that ROS could interact with many biological macromolecules, such as lipids, proteins and DNA, causing structural changes, functional abnormalities, and diabetic complications^4,5^. Clinically, the complications were associated with organ damage, mainly focused on liver, kidney, pancreas and heart^21^. Therefore, antioxidants may play important roles in preventing DM and its complications by directly interfering with the generation of ROS. Currently, the protective effects on the kidney in STZ-treated mice, which are accompanied by the antioxidant activities of EPS and its two hydrolysates (EEPS and AEPS) from *H. marmoreus*, were investigated using serum and tissue parameters.

The kidney is an organ that plays a vital role in the glucose metabolism of diabetic animals. Three serum parameters, including the BUN, CRE and ALB levels were always used for clinical kidney examination. BUN, as the first endogenous substance generated by the decomposition of liver proteins, was excreted by the filtration of the glomerulus. CER, as the byproduct of creatine and phosphocreatine catabolism, was endogenously produced and released into the body fluids^22^. In addition, the ALB levels could also be an indication of nephropathy, reflecting increments of the glomerular filtration rate because the damaged kidney could expedite excessive plasma protein filtering into the urine^11^. Previous literature has indicated that STZ could directly induce hyperglycemia, owing to its selective toxicity on pancreatic β-cells, resulting in the disordered secretion of insulin^23^. Huang *et al*.^24^ indicated the pancreatic protection of polysaccharides may be attributed to its anti-diabetic activity.

As a metabolic disorder, DM was characterized not only by increased glucose levels but also by dysregulation of lipid profiles. In the cholesterol families, the HDL-C could carry the cholesterol ester from the peripheral tissues to the liver by the “reverse cholesterol transport” pathway during blood circulation, inhibiting the risk of atherosclerotic cardiovascular diseases. However, the other two lipoproteins of LDL-C and VLDL-C could accelerate the risk factors for coronary heart disease^8^. In addition, higher TC and TG levels could be a risk for atherosclerotic plaque lesions, attributing to their pathological accumulation in the blood vessel walls^11^. In this work, the administration of three polysaccharides (EPS, EEPS and AEPS) from *H. marmoreus* led to the improvement of TC, LDL-C and VLDL-C and the decline of HDL-C, providing clear evidence that the polysaccharides had potential protective effects on organs.

Furthermore, the activities of the antioxidant enzymes (SOD, GSH-Px, CAT and T-AOC), and lipid contents (LPO and MDA) were observed to investigate the protective effects of STZ on organ damage against oxidative stress. Sabir *et al*.^25^ reported that SOD and GSH-Px, the important free radical scavenging enzymes in the first line of defense against oxidative injury, were responsible for the detoxification of deleterious oxygen radicals and played important roles in protecting the cell from oxidative damage. Meanwhile, the decrease in the SOD activity could lead to an excess of superoxide anion and hydrogen peroxide (which in turn generated hydroxyl radicals) in biological systems, resulting in the initiation and propagation of lipid peroxidation. The decomposition of hydrogen peroxide could be catalyzed into less-reactive gaseous oxygen and water molecules by CAT^26^. In addition, the GSH-Px, which played a vital role mainly in the detoxication and metabolism as a cofactor or a substrate for some enzymes, could catalyze the reduction of H_2_O_2_ into H_2_O and O_2_^25^. Thus, GSH-Px may be measured as a common marker of free radical damage in protecting tissues from oxidative stress.

In the free radical hypothesis, these reactive radicals could react with polyunsaturated fatty acids in cell membranes, leading to lipid peroxidation and dysfunction of membranes and subsequently resulting in tissue injury. Simultaneously, excess free radical could also be generated during abnormal lipid peroxidation^11^. Can *et al*.^27^ indicated that the *in vivo* organ damage caused by diabetes was probably due to free radicals produced by lipid peroxidation. Reed^28^ had also reported that the content of MDA, an end product of lipid peroxidation, may provide a convenient index of lipid peroxidation.

The biological functions, including antioxidant properties, were mainly associated with their monosaccharide compositions, molecular weights, bond types, and so on^26^. In the present work, all the results indicated that EEPS exhibited potentially superior protective effects on kidney damage compared to EPS and AEPS. Based on the monosaccharide analysis, only EEPS contained L-rhamnose, indicating that the L-rhamnose may play important role in maintaining protective effects against kidney damage. Furthermore, the NMR analysis of EEPS agreed with preliminary test results. According to previous studies^29^, chemical shifts correspond to C2 and C6. These observations were in accordance with other studies^19^. The analysis of ^13^C NMR spectra showed that the polysaccharides from *H. marmoreus* contained a high level of glucose, and the possible mechanism may be due to the type of homopolysaccharides (glucans). The results indicate that the superior effects on STZ-induced kidney damage were responsible for these special characteristics. Meanwhile, similar results of *Grifola frondosa*^19^ and *Macrocybe gigantea*^30^ could confirm our conclusions. The characteristic changes coincided with the shape changes in the polysaccharides. The SEM of EPS, EEPS and AEPS as well as their derivatives at magnifications of 25000 demonstrated these results. The results showed that the different extraction methods induced different physical changes in size and shape. The SEM analysis demonstrated that EPS was relatively regular and homogeneous in shape, while the surface of EEPS and AEPS was rough and had an irregular shape, with the possible mechanism potentially owing to the branches and network structures of the polysaccharides. Previous literature has indicated that the surface topography of a polysaccharide may be influenced by different methods of extraction, purification, and preparation^31^. In the present work, the difference in the SEM of the three polysaccharides may be that the polysaccharide properties had been well changed by enzymatic hydrolysis, showing superior physicochemical properties with good water solubility, high stability, safety and a lack of toxicity^32^. Furthermore, the main advantages of enzymatic hydrolysis were the high selectivity and substrate specificity, enabling products with well-defined and stereospecific structure^33^. In agreement with these results, similar conclusions were also reported by Zhao *et al*.^8^ for *Termitomyces albuminosus*, Jia *et al*.^34^ for *Cordyceps militaris* fruit bodies, and Yang *et al*.^35^ for *Phoma herbarum*. Previous and present conclusions demonstrated that the extraction methods played important role in maintaining the biological properties due to their unique effects on influencing the shape and structure of the substances.

## Conclusions

In the present work, EPS and its two EEPS and AEPS hydrolysates were isolated and characterized from *H. marmoreus*, and the protective effects on the kidney in diabetic mice induced by STZ were investigated. The results provided evidence that EPS, EEPS, and AEPS could effectively protect organs against STZ toxicity and could be used as potentially natural and functional ingredients in the prevention and alleviation of DM and its complications.

## Materials and Methods

### Chemicals and strain

The diagnostic kits for assaying SOD activity, GSH-Px activity, CAT activity, T-AOC activity, and LPO and MDA content were purchased from Nanjing Jiancheng Bioengineering Institute (Nanjing, China). Snailase and STZ were purchased from Sigma Chemicals Company (St. Louis, USA). Blood glucose test strips were purchased from Sinocare Biosensing Corporation Limited (Changsha, China). All other chemicals were of analytical grade and purchased from local chemical suppliers.

### Culture media and conditions

The *H. marmoreus* strain used in this experiment was provided by the Shandong Agricultural Academy of Sciences (Shandong, China) and maintained on a potato dextrose agar (PDA) slant at 4 °C. The liquid culture (1 L) with natural pH was composed of 20 g of glucose, 3 g of peptone, 4 g of yeast extract, 1 g of KH_2_PO_4_ and 1 g of MgSO_4_. A 0.5-cm^2^ portion of the agar plate culture was sliced from the slant for inoculating the seed culture medium. Seeding cultivation in liquid media was cultured in a 1 L filter flask containing 600 mL of potato dextrose broth at 25 °C for 24 h without shaking and then shaken on a rotary shaker (160 rpm, Anting, Shanghai, China) for 7 days. The submerged fermentation was carried out in a 100-L fermentation tank (Xianmin, Luoyang, China) for 14 days (temperature 25 °C and pH 7) with the previous liquid culture media.

### Preparation of EPS

The fermentation broth of *H. marmoreus* was centrifuged at 3000 rpm for 15 min, and supernatant liquid was collected. After concentrating fivefold under vacuum, the supernatant was mixed with 3 volumes of 95% ethanol (v/v), standing at 4 °C for 24 h. After centrifugation at 3000 rpm for 15 min, the precipitate was collected and purified by the Sevag method^36^. Then, the purified polysaccharides were considered as EPS by lyophilization, and vacuum freeze drying (Labconco, USA) with the contents determined by phenol-sulfuric acid, using glucose as the standard^37^ with the EPS yields of 1.44 ± 0.57 g/L.

### Enzymatic and acidic degradation

The EEPS and AEPS were separately prepared according to the methods reported by Yang, *et al*.^35^ with slight modifications.

The enzymatic hydrolysis of EPS was carried out in snailase solution (1%, dissolved in sodium acetate buffer) depending the following conditions with a ratio of 1:4 (materials to snailase solution, w/v), extraction temperature of 40 °C, pH of 6, and extraction time of 5 h^35^. The final EEPS was obtained by concentration, deproteinzation^36^, and lyophilization.

The EPS in the test tube (18 mm × 180 mm) was hermetically hydrolyzed with H_2_SO_4_ (1 M, 1:20, w/v) in boiled water for 8 h. After centrifugation at 3000 rpm for 15 min, the supernatant was neutralized with NaOH solution (2 M). The AEPS was consecutively extracted by concentration, deproteinzation^36^ and lyophilization.

### Animal experiments

The experiments were performed and approved by the institutional animal care and use committee of Shandong Agricultural University and in accordance with the Animals (Scientific Procedures) Act of 1986 (amended 2013). The Kunming strain of mice, weighing 20 ± 0.2 g, were purchased from the Taibang Company (Taian, China) and housed in stainless steel cages under controlled conditions (temperature 23 ± 2 °C, lights on 12 h every day). After three days to allow the mice to get acclimated, diabetes was induced by an intraperitoneal injection with STZ (80 mg/kg, freshly prepared in citrate buffer solution, 0.1 M, pH 4.5) for three successive days (once every day). The mice were considered diabetic when the tail-vein GLU levels were higher than 13.3 mM^14^. Successful diabetic mice were randomly divided into eleven groups of ten mice each, including one MC treated with distilled water; one PC group that received glibenclamide (20 mg/kg); and nine dosage groups treated with EPS, EEPS and AEPS at three different doses (200, 400 and 800 mg/kg), while another ten mice in the NC groups (normal mice without STZ-intervention) were treated with distilled water. The glibenclamide, polysaccharide and distilled water gavages were processed with a syringe daily, and the experiments lasted for 14 days. The body weights of the mice were measured every day. At the end of the experiment, the GLU levels from the tail vein were monitored by blood glucose test strips after fasting overnight, and the mice were sacrificed under ether anesthesia.

The serum in all the mice was collected by centrifugation (14000 rpm, 4 °C) for serum analysis. The serum levels of BUN, CRE, ALB, TC, TG, HDL-C, VLDL-C and LDL-C were measured using an automatic biochemical analyzer (ACE, USA).

The right kidney was randomly isolated from one mouse in each group and freshly immersed in PBS buffer (10% formalin, pH 7.4) for over 24 h and embedded in paraffin. Thin sections (4–5 µm thickness) were prepared using a microtome and stained with hematoxylin-eosin. Each section was photographed under a microscope to show the histopathological changes (×600 magnifications).

The other kidneys were rapidly weighed and homogenized (1:9, w/v) in 0.2 M phosphate buffer (4 °C, pH 7.4). The organic supernatants were collected by centrifugation (14000 rpm, 10 min), and the SOD, GSH-Px, CAT, and T-AOC activities, as well as the MDA and LPO contents, were determined by commercial reagent kits according to their instructions. The tissue index was calculated using the following equation: (tissue weight/body weigh) (g/100 g body weight).

### Acute toxicity assay

An acute toxicity study was performed by the method of Chao *et al*.^38^ Twenty Kunming strain mice were randomly divided into four groups (five in each group). In the control group, mice were given free access to diet and water, while in the experimental groups, mice were orally given three polysaccharides at the ultimate dosage of 4000 mg/kg. The mice were observed continuously for the first 24 h for any gross behavioral changes and toxic symptoms, as well as for mortality in the first 48 h.

### Characterization of EPS, AEPS and EEPS

The molecular weights and homogeneities were determined by HPLC on an HPLC system (Shimadzu LC-2010AT, Japan) equipped with an Atlantis C18 column (250 mm × 4.6 mm × 5 µm) and a refractive index detector. Deionized water was used as the mobile phase with a flow rate of 1 mL/min, and the column temperature was maintained at 30 °C. A series of standard dextrans were used to make the calibration curve. The molecular weights were analyzed by Agilent GPC software.

The morphological features of EPS, EEPS and AEPS were analyzed by SEM analysis (S-4800, FE-SEM, Hitachi High-Technologies, Japan). The dried powder of the polysaccharides was affixed on a glass slide and coated with gold powder to make the samples conductive. The images with a magnification of 25000 × were taken with an accelerating voltage of 10 kV.

Monosaccharide composition was determined by GC (GC-2010, Shimadzu, Japan) equipped with a capillary column of Rtx-1 (30 mm × 0.25 mm × 0.25 µm) using a previously published method^39^. Composition identification was processed by comparison with standard monosaccharides (mannose, rhamnose, glucose, galactose, arabinose, ribose, and xylose). The relative molar ratios were calculated by the area normalization method according to the chromatogram.

The dried polysaccharides samples (1 mg) were mixed with KBr powder (100–200 mg), and then the mixture was ground in a mortar under an infrared lamp to prevent the air-slake of KBr. After tableting, spectra were recorded on a 6700 Nicolet Fourier transform-infrared spectrophotometer (Thermo Co., Madison, WI, USA) within the range from 4000 to 400 cm^−1^.^40^

^13^C and ^1^H NMR spectroscopy experiments were conducted using a 700-MHz Varian Mercury 2010 Magneto Oxford spectrometer at 60 °C, and the samples were dissolved in dimethyl sulfoxide (DMSO).

### Statistical analysis

All data were processed and analyzed using SAS and expressed the means ± standard deviation (SD). Two-way analysis of variance (ANOVA) followed by post-hoc Tukey’s tests were performed to statistically test the differences, and *P* < 0.05 was considered significant.
